# A combined structural and biochemical approach reveals translocation and stalling of UvrB on the DNA lesion as a mechanism of damage verification in bacterial nucleotide excision repair

**DOI:** 10.1016/j.dnarep.2019.102746

**Published:** 2019-11-06

**Authors:** Marcin Jaciuk, Paolo Swuec, Vineet Gaur, Joanna M. Kasprzak, Ludovic Renault, Mateusz Dobrychłop, Shivlee Nirwal, Janusz M. Bujnicki, Alessandro Costa, Marcin Nowotny

**Affiliations:** a Laboratory of Protein Structure, International Institute of Molecular and Cell Biology, Trojdena 4, Warsaw, 02-109, Poland; b Molecular Machines Laboratory, The Francis Crick Institute, London, NW1 1AT, UK; c Laboratory of Bioinformatics and Protein Engineering, International Institute of Molecular and Cell Biology, Trojdena 4, Warsaw, 02-109, Poland; d Institute of Molecular Biology and Biotechnology, Faculty of Biology, Adam Mickiewicz University, Umultowska 89, Poznan, 61-614, Poland

**Keywords:** Prokaryotic nucleotide excision repair, UvrA, UvrB, UvrC, DNA repair

## Abstract

Nucleotide excision repair (NER) is a DNA repair pathway present in all domains of life. In bacteria, UvrA protein localizes the DNA lesion, followed by verification by UvrB helicase and excision by UvrC double nuclease. UvrA senses deformations and flexibility of the DNA duplex without precisely localizing the lesion in the damaged strand, an element essential for proper NER. Using a combination of techniques, we elucidate the mechanism of the damage verification step in bacterial NER. UvrA dimer recruits two UvrB molecules to its two sides. Each of the two UvrB molecules clamps a different DNA strand using its β-hairpin element. Both UvrB molecules then translocate to the lesion, and UvrA dissociates. The UvrB molecule that clamps the damaged strand gets stalled at the lesion to recruit UvrC. This mechanism allows UvrB to verify the DNA damage and identify its precise location triggering subsequent steps in the NER pathway.

## 1 Introduction

Nucleotide excision repair (NER) is a major pathway that mediates DNA repair in cells. It is present in all domains of life and can detect and correct various DNA modifications that vary in size and chemical structure [1,2]. There are two varieties of NER. Global genome repair removes damage throughout the entire genome, particularly from non-transcribed DNA. Transcription-coupled repair preferentially repairs transcribed strands of genes and requires RNA polymerase that stalls at the lesion site [3].

Nucleotide excision repair was first discovered in bacteria in studies that investigated the removal of ultraviolet light-induced DNA lesions [4,5] and it was later reconstituted in *vitro* [6]. Three key proteins mediate bacterial NER: UvrA, UvrB and UvrC [2]. UvrA dimer detects DNA damage and subsequently two UvrB molecules are loaded on the DNA by interacting with UvrA [7]. UvrB verifies the presence of damage and UvrA dissociates from the complex. Then, UvrC, which contains two nuclease domains, binds UvrB and executes a cut on each side of the lesion. UvrD helicase removes the excised DNA fragment, which is replaced with new DNA by polymerase I. The localization of the DNA damage can be performed either by UvrA alone using three-dimensional search or by the UvrA—UvrB complex performing both three-dimensional search and one-dimensional sliding on the DNA [8]. Another study implicated that dimeric UvrA alone scans the genome and recruits UvrB only after recognizing the damage [9]. An important element of the NER mechanism is nucleotide tri-phosphate (ATP) binding and hydrolysis by UvrA [10]. The role of this reaction in DNA damage detection is quite complex and has been recently characterized using fast kinetics methods [11].

Over the years, extensive structural information has been obtained for the components of bacterial NER. Important structures include atomic models of DNA-free form of UvrA [12,13], UvrA—DNA structure [14], several apo structures of UvrB [15–17] as well as a structure of *Bacillus caldotenax* UvrB bound to a short fragment of the DNA [18]. UvrA-UvrB interactions have been characterized based on the crystal structure of a complex of interacting domains from UvrA and UvrB [19] and complex of UvrA-UvrB complex in absence of the DNA [7]. The latter work implied that UvrB is loaded away from the DNA lesion and must translocate to reach it. A key structure which still needs to be determined is that of a complex of UvrA, UvrB and DNA.

Damage verification by UvrB remains a critical determining step in NER. UvrA does not confer absolute specificity for damaged DNA. In fact, it binds damaged DNA with only a slightly higher affinity than non-damaged DNA [14,20] and localizes only the approximate region where damage occurred. Symmetry of UvrA dimer is reflected in its DNA interactions [14]. So, it can effectively probe the conformation of the DNA helix but cannot distinguish which DNA strand is damaged. After the preliminary screen by UvrA, UvrB verifies the presence of the DNA modification, discriminates the damaged from undamaged DNA strands and precisely localizes the site of DNA lesion.

Since incisions by UvrC require high precision and should occur only in the damaged strand [21], the damage verification step by UvrB that triggers UvrC recruitment is critical. However, several key elements of this mechanism remain unclear. In particular, the mechanism through which UvrB converts the initial symmetric and approximate recognition of the lesion to a strand-specific, precise localization of the damage site remains to be determined. UvrB possesses an important β-hairpin element which clamps one of the DNA strands and is critical for DNA binding by the protein [17]. A key structural question concerning this damage verification process is whether the damaged DNA strand bound by UvrB lies either under or on top of the β-hairpin element [21]. This positioning remains a critical and contested topic [6,22–24]. The placement of the damaged strand relative to the hairpin imposes the topology of DNA binding by the UvrA—UvrB complex. Different DNA placement will result in distinct damage verification mechanisms.

We sought to elucidate the mechanism that underlies the DNA damage verification step in bacterial NER. Using biochemical and structural studies alongside computational modeling we establish the architecture of the UvrA—UvrB—DNA complex, which was not previously described. Based on this structure we propose a mechanism of damage strand identification by UvrB. We validate this mechanism using biochemical experiments.

## 2 Results

### 2.1 Purification of UvrA—UvrB—DNA complex

To understand the mechanism that drives the damage verification step in bacterial NER, we prepared a stable UvrA—UvrB—DNA complex for structural studies. We first performed extensive screening of UvrA and UvrB proteins from five different bacterial and archaeal species in combination with several DNA substrates. These substrates contained a centrally located fluorescein-modified thymine residue or two modified thymines separated by 4 bp and located in opposite strands. We prepared the latter DNAs from a self-annealing palindromic oligonucleotide. Previously, doubly-modified DNAs were successfully used to crystallize a *Tm*-UvrA—DNA complex [14]. In that study we proposed that the presence of two modified residues promoted UvrA binding in a better-defined register on the DNA, leading to a more homogeneous nucleoprotein assembly. All DNA substrates contained a central 32 bp double-stranded region corresponding to the DNA region covered by UvrA [14]. To permit UvrB binding, this central part was flanked by either 10 bp double-stranded regions or 10 nt overhangs on the 5′ or 3′ end.

Various combinations of proteins and DNA substrates were tested for UvrA—UvrB—DNA complex reconstitution either through size-exclusion chromatography coupled with multi-angle light scattering (SEC-MALS) or glycerol gradient purification and visualization by negative stain electron microscopy (EM). We found that the addition of ADP and Mg^2+^ ions promoted complex formation. Only one complex appeared sufficiently stable and compositionally homogenous for single-particle EM analysis. This complex comprised *T. maritima* (*Tm*) UvrA, *Thermus thermophilus* (*Tt*) UvrB and DNA with two modified thymines and single-stranded 10 bp 5′ overhangs (Supplementary Figure S1a). For *Tm*-UvrA—*Tt*-UvrB—DNA complex, the SEC elution profile comprised several peaks (Supplementary Figure S1b). The most prominent peak contained both UvrA and UvrB components as confirmed by SDS-PAGE analysis and the DNA as indicated by the Abs260/Abs280 ratio. The molecular weight (MW) of the species in the main peak measured by MALS was 395.8 ± 2.8 kDa, corresponding to assembly of one UvrA dimer, two UvrB monomers, one DNA molecule and six ADP molecules (theoretical MW of 387.9 kDa; *Tm*-UvrA (dimer): 205.8 kDa, *Tt*-UvrB: 76.2 kDa, DNA: 26.4 kDa). This implies UvrA_2_—UvrB_2_—DNA complex stoichiometry, which is in agreement with earlier work in which two UvrB molecules were detected in the pre-incision complex [25]. The stability of the UvrA—UvrB—DNA complex was verified by pooling the peak fractions and re-running over SEC-MALS resulting in one single peak with unvaried retention time (data not shown). We next confirmed that a system composed of *Tm*-UvrA, *Tt*-UvrB and *Tm*-UvrC is functional in a NER reaction (Supplementary Results, Supplementary Figure S1c). In summary we successfully reconstituted a stable and functional Uv-rA—UvrB—DNA complex.

### 2.2 Single-particle reconstruction

**Fig. 1:**
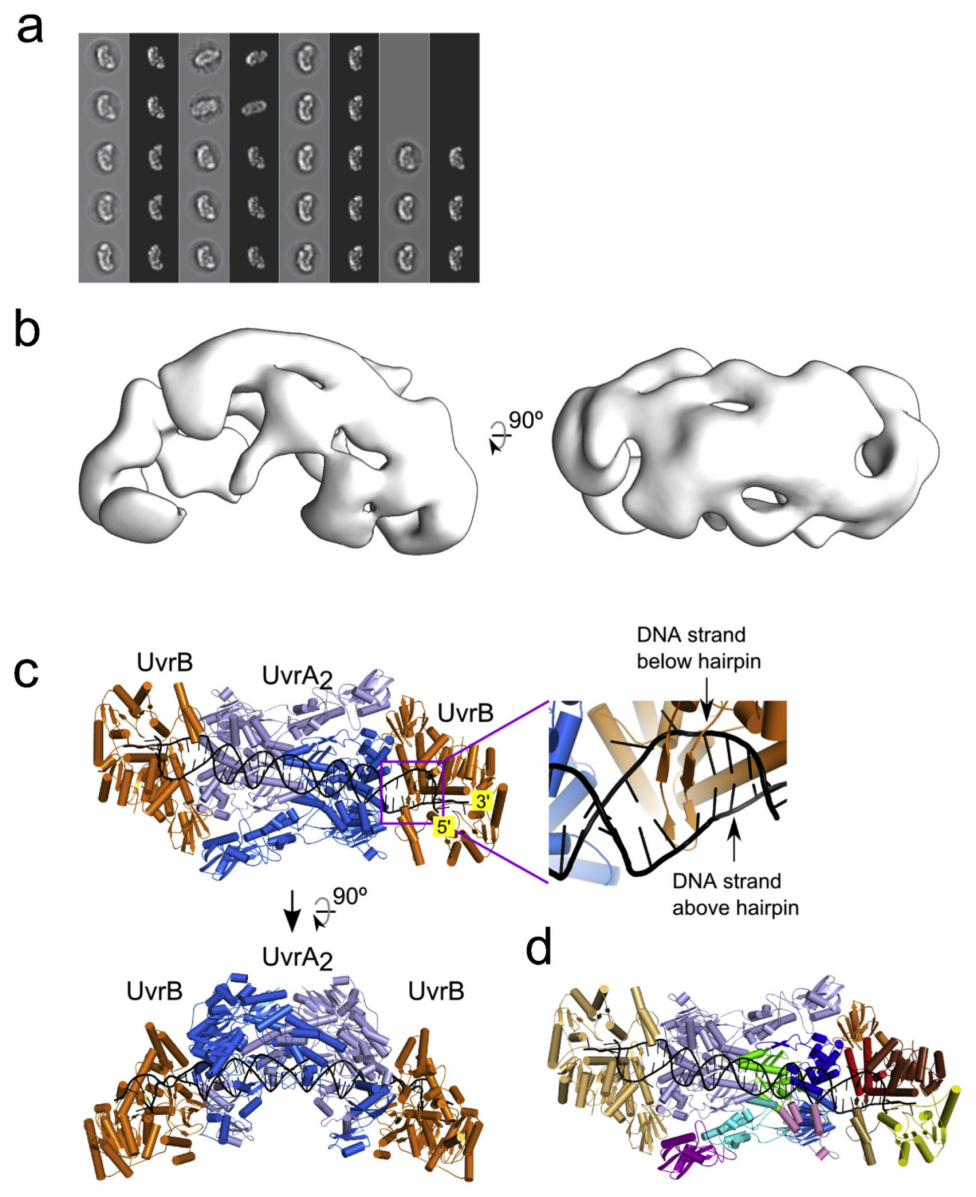
Single-particle negative-stain electron microscopy reveals the UvrA—UvrB—DNA complex structure. (a) Match between reference-free 2D class averages (gray back-ground) and forward-projections of the 3D structure. (b) Surface rendering of the negative-stained electron microscopy structure refined to 25.5 Å resolution. (c) Model of UvrA_2_—UvrB_2_—DNA complex (orthogonal views). UvrA dimer shown in two shades of blue. UvrB shown in orange. DNA shown in black. The inset shows a close-up view of the β-hairpin in the UvrB structure that clamps one strand of modeled DNA (based on PDB ID 2FDC) [18]. (d) Structural model colored according to protein domains. One entire UvrA subunit and one UvrB subunit are in pale blue and pale orange, respectively, while the other subunits are colored. UvrA, green − ATP-binding I, cyan − signature I, marine − ATP-binding II, blue − signature II, purple − UvrB-binding, pink − insertion domain. UvrB, brown − 1a, red − 1b, orange − 2, yellow − 3, sand − 4.

To characterize the architecture of the UvrA—UvrB—DNA assembly and derive testable functional predictions, we performed negative-stain electron microscopy (EM) single-particle reconstruction of the reconstituted complex. In the negative-stain micrographs, we found elongated particles that showed a mono-dispersed and homogeneous distribution (Supplementary Figure S2). We extracted 23,547 particles from 423 micrographs and refined the three-dimensional (3D) structure based on 10,523 particles selected after cleaning by two-dimensional (2D) and 3D classification (Fig. 1, Supplementary Figure S2). We estimated the resolution of the 3D structure (25.5 Å) using the gold-standard Fourier shell correlation with a 0.143 cutoff. Although we did not impose any symmetry at any stage during structure determination, the structure showed marked, two-fold symmetric character. We note that negative stain EM generally does not allow visualization of nucleic acids (for example see [26]), explaining why no DNA density can be visualized in our reconstruction.

Although the limited resolution of our reconstruction does not allow flexible fitting of sub-domains, it is sufficient for docking of entire models of UvrA—UvrB complex. We first compared our EM structure with the DNA-free UvrA_2_—UvrB_2_ complex crystal structure (Protein Data Bank [PDB] ID 3UWX) [7]. We performed docking using the *fit in map* command in UCSF Chimera [27] by comparing the EM map and a modeled electron density map derived from the atomic structures and filtered to 25.5 Å resolution (Supplementary Figure S3a, b). This comparison revealed a poor fit between the EM map and crystal structure, with unoccupied densities and several UvrA and UvrB domains residing outside the EM map. As a result, a cross-correlation coefficient between EM and X-ray structures was only 0.82. This is in agreement with the original analysis that indicated that the conformation observed in the 3UWX crystal structure is not compatible with the binding of damaged DNA [7].

### 2.3 A molecular model of UvrA—UvrB—DNA complex

We constructed a model of the UvrA—UvrB—DNA complex using ample available structural information on the components and their subcomplexes (Supplementary Figure S4), with the exception of our EM data, which were saved for independent validation of the modeling results. Given the dimeric nature of UvrA, the available structural data and results from our SEC-MALS experiments, we assumed that UvrA—DNA recruits two UvrB molecules at the same time. We used the following structures: *T. maritima* UvrA (*Tm*-UvrA) in a complex with DNA (PDB ID 3PIH) [14], a complex of the interacting domains from UvrA and UvrB from *Geobacillus stearothermophilus* (PDB ID 3FPN) [19], and the *B. stearothermophilus* UvrA (*Bs*-UvrA) structure (PDB ID 2R6F) [12]. We built a homology model of *T. maritima* UvrB (*Tm*-UvrB) using the structure of *B. caldotenax* UvrB (*Bca*-UvrB) in complex with DNA (PDB ID 2FDC) [18]. This homology model also included the C-terminal helical domain IV of UvrB (PDB ID 1QOJ) [28], which is absent in the *Bca*-UvrB−DNA structure. The modeling is described in detail in the Materials and Methods section. Briefly, we applied PyRy3D software for macromolecular modeling using restraints (http://genesilico.pl/pyry3d/). To account for conformational flexibility during Uv-rA—UvrB—DNA complex assembly, we defined independent structural modules in UvrA and UvrB (see Methods for more details) to allow their movement during the simulation leading to the complex formation. We determined the orientation of one UvrB molecule bound to a DNA fragment relative to the UvrA dimer bound to another DNA fragment by performing a global search initiated from random positions of all domains in space. We used independent simulations to determine the length of the DNA sequence between UvrA- and UvrB-bound sites that optimized interactions between the proteins in complex. To obtain the final model, we optimized the local conformation of the protein and DNA chains by flexible refinement and generated a symmetrical structure with two identical copies of the UvrB molecule bound to both ends of the UvrA dimer.

We show the final model in Fig. 1c. The UvrA dimer that interacts with DNA is flanked by two UvrB molecules. Each UvrA-UvrB interface comprises two contact points. The first interface possesses contacts between the UvrB-binding domain of one protomer from the UvrA dimer and domain 2 from UvrB (Fig. 1d). The second interface arises between the signature II domain of the second protomer of the UvrA dimer and domain 1b of UvrB. These interfaces had been identified in the UvrA—UvrB crystal structure and verified in mutagenesis studies [7]. In addition, domain IV of UvrB in our model lies in close proximity to the DNA-binding domain of UvrA, suggesting that these domains can form additional UvrA-UvrB contacts. This prediction is consistent with biochemical data demonstrating that domain IV mediates the interaction between UvrA and UvrB [29].

**Fig. 2:**
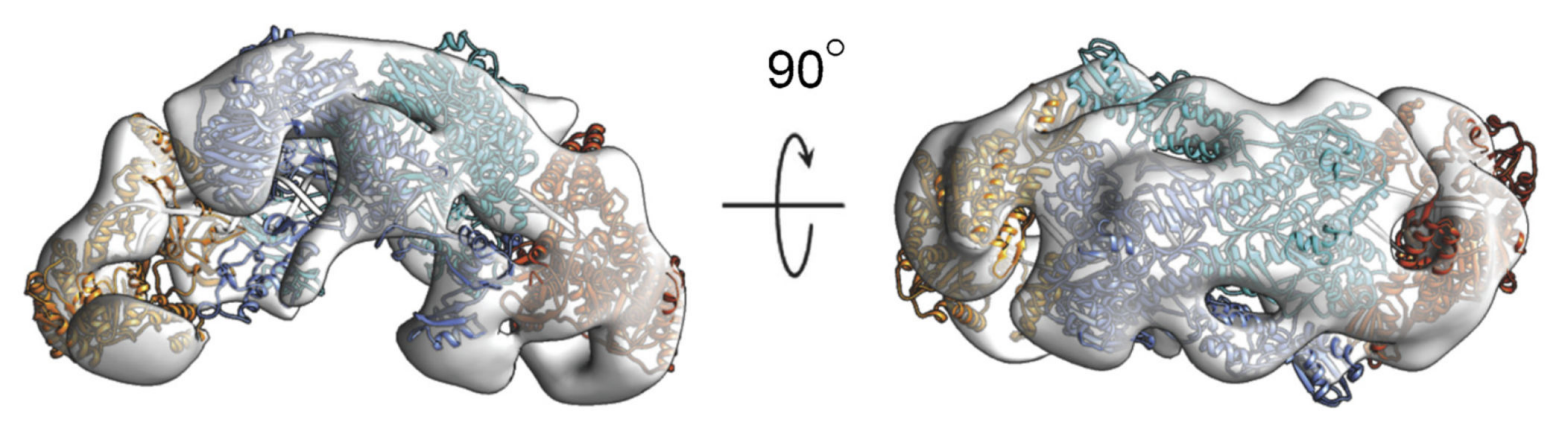
Fit of the theoretical model of UvrA2—UvrB2—DNA complex to an independently obtained EM map (two views).

While the overall subunit arrangement in our UvrA—UvrB—DNA model resembles the known DNA-free UvrA_2_—UvrB_2_ crystal structure, key structural elements of the two models differ. Compared with the UvrA_2_—UvrB_2_ crystal structure, our model yields a better fit with our negative-stain EM structure with a CCC improving from 0.82 to 0.9 (Fig. 2, Supplementary Figure S3c-e). We note that this difference is meaningful, because it involves modeling and EM experiments, which were performed independently. The differences between our model and the UvrA_2_—UvrB_2_ crystal structure mostly arise from different UvrA dimer interactions (Supplementary Figure S5). The dimer arrangement in our model is the same as in the crystal structures of UvrA proteins from *T. maritima* (complex with DNA, PDB ID 3PIH) [14], *B. stearothermophilus* (PDB ID 2R6F) [12] and *M. tuberculosis* (PDB ID 3ZQJ) [30]. The differences between the UvrA_2_—UvrB_2_ crystal structure and our model likely occur, since the former is not compatible with the binding of damaged DNA [7]. Therefore, we conclude our model represents the configuration of UvrA_2_—UvrB_2_—damaged DNA complex.

### 2.4 Biochemical validation of the model and insight in the second step of bacterial NER

**Fig. 3:**
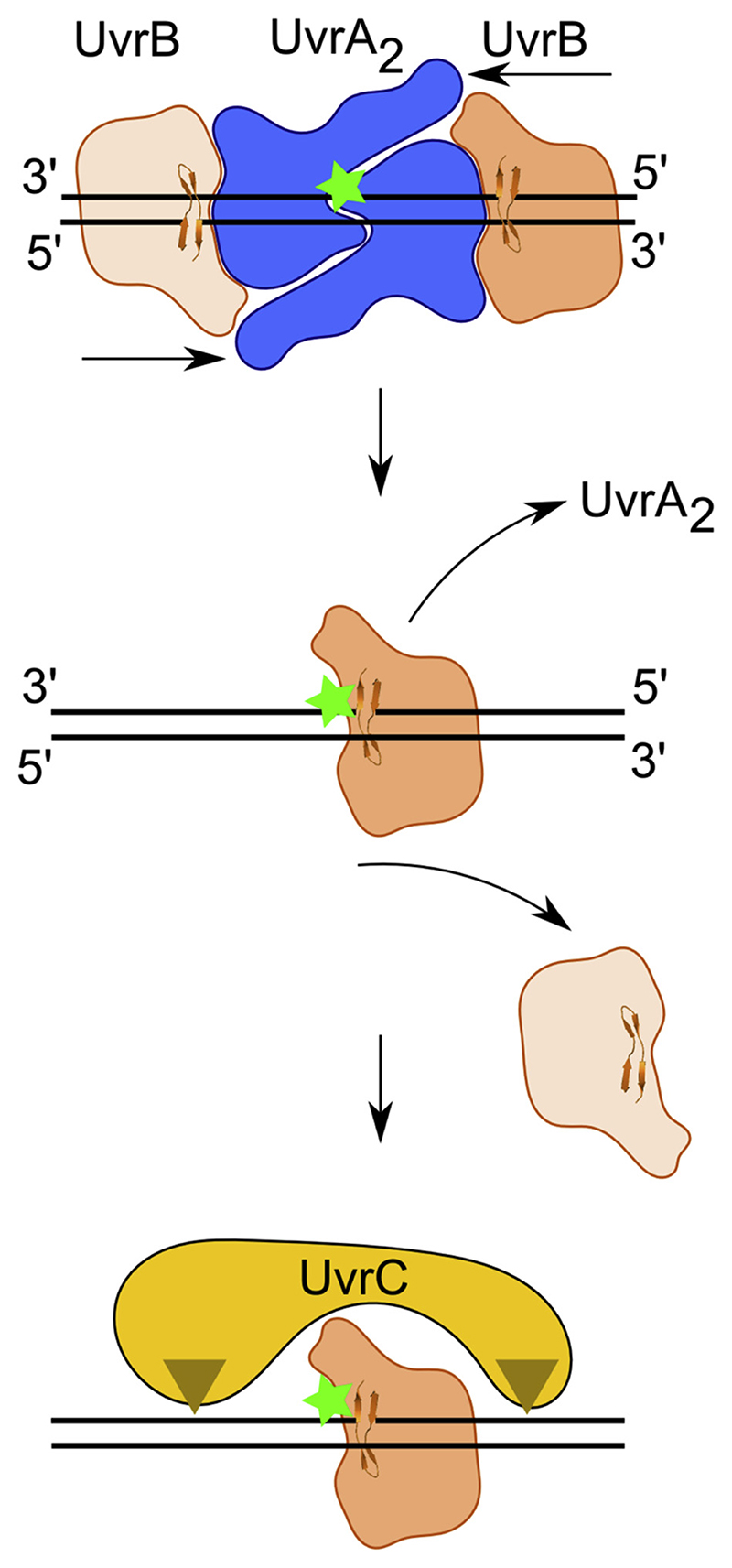
Model of bacterial NER. The UvrA dimer bound at the site of DNA modification recruits two UvrB molecules. Each UvrB molecule clamps a different DNA strand under the β-hairpin element (upper panel). Both UvrB molecules then translocate toward the lesion with 5′ to 3′ polarity on the strand under the hairpin. The UvrB molecule that clamps the modified strand will stall at the lesion (green star indicates the site of DNA modification) and the other UvrB molecule (light orange) will dissociate (middle panel). The stalled UvrB recruits UvrC double nuclease (shown in yellow), which makes two incisions indicated with triangles. Note: the top strand is shown in a 3′ to 5′ direction to match the representation of the UvrA_2_—UvrB_2_—DNA model in previous figures.

A key finding from our theoretical modeling and EM reconstruction analyses is that the DNA strand clamped under the β-hairpin of the UvrB protein connects to the 5′ terminus of the double-stranded portion of the DNA bound by UvrA. Therefore, each UvrB protein in the complex clamps a different strand of the DNA and each strand is clamped upstream from the damaged region. In agreement with a previous proposal [7], this implies that both UvrB molecules translocate in 5′ to 3′ direction on the clamped strand toward the lesion. This leads to a mechanistic model in which the UvrB molecule, which clamps the damaged strand stalls at the lesion, remains stably bound and subsequently recruits UvrC. The second UvrB molecule that clamps the unmodified strand instead dissociates (Fig. 3). A possibility of such stalling of UvrB at the lesion has been previously discussed [18]. This mechanism provides rationale for how bacterial NER determines which DNA strand is damaged − the information not provided by the initial symmetrical damage detection by UvrA [14].

**Fig. 4:**
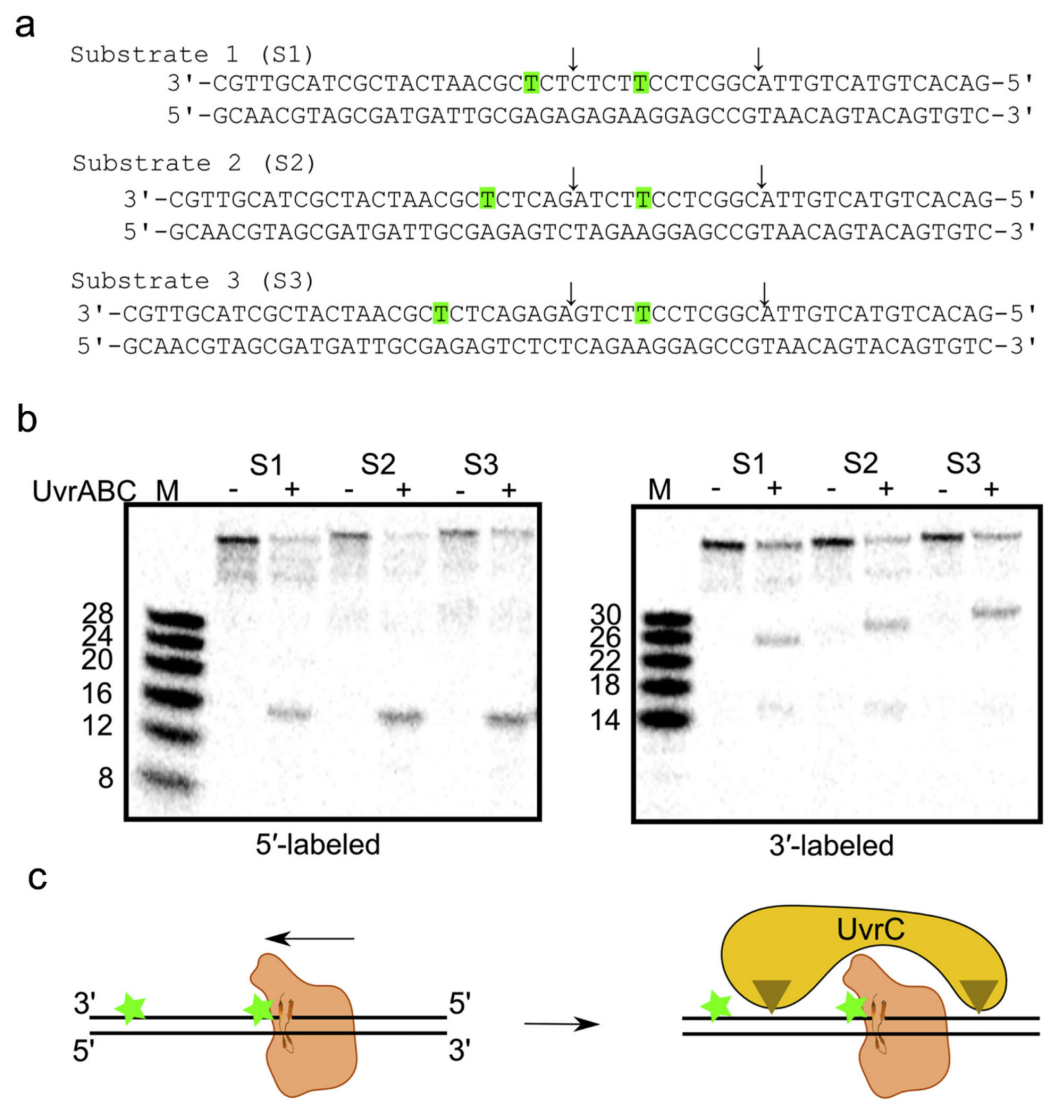
Incision assay with doubly modified DNA oligonucleotide. (a) The DNA substrates used in this experiment. The residue bases in green are modified with fluorescein. Sites of observed cuts indicated with arrows. Note: the sequence of the modified strand is shown in a 3′ to 5′ direction to match the orientation in Fig. 3. (b) Incision of substrates shown in (a) by reconstituted bacterial NER machinery (*T. maritima* UvrA, UvrB, and UvrC). Radiolabeled substrate at the 5′ terminus or 3′ terminus. Note: 3′-labeled products migrate more slowly than expected due to the presence of the second modified thymine (see Materials and Methods). Experiment replicated four times. (c) Schematic of the experiment (colors as in Fig. 3).

We verified the structural and mechanistic models biochemically. Previous results indicated that UvrB must approach the lesion from the 5′ end of the damaged strand [31]. In agreement with that, our model implies that damage verification requires that UvrB translocates in the 5′ to 3′ direction on the damaged strand and stalls at the lesion. We reasoned that if two modifications occur in one strand, then the modification located closer to the 5′ end would first stall UvrB translocation and prevent recognition of the modification located toward the 3′ end. So, the UvrABC machinery will specifically recognize only the modification located closer to the 5′ end. To test this hypothesis, we prepared three DNA substrates, each containing two fluorescein-modified thymines in one strand (Fig. 4a). The substrates varied in the distance between the two modified nucleotides (6, 9, 12 bp) and consequently in length. These oligonucleotides were radioactively labeled on either the 5′ or 3′ end and mixed with *Tm*-UvrA, *Tm*-UvrB, and *Tm-*UvrC in the presence of ATP to reconstitute the incision reaction. In agreement with our prediction, in all three substrates *Tm*-UvrC cleavage occurred on the two sides of the modification located closer to the 5′ end of the modified strand (Fig. 4b). These results are in agreement with our model and show that the damage is searched by UvrB from the 5′ end of the damaged strand (Fig. 4c).

We note a minor band present for the reaction with a 3′-labeled substrates S1 and S2. The size of this band is the same size in both S1 and S2 reactions (16 nucleotides). This implies that it results from cleavage on the 3′ side of the second lesion. Notably, no corresponding cleavage on the 5′ side of the second lesion is observed indicating that there is no bona fide repair of the second modification by the UvrABC system, in contrast to the processing of the first modification. The minor 16 nt band can result from secondary reactions in which the first lesion is removed which exposes an entry point for the processing of the second lesion. Another possibility is that this band is a result of a minor side reaction that could have resulted from bypassing the first damaged site from 5′ end. UvrA can bind the DNA in multiple registers, also in non-modified regions of the DNA. The additional band can result from rare situations in which the UvrA is located in a register which would load UvrB beyond the 5′ modification.

### 2.5 Chemical cross-linking

**Fig. 5:**
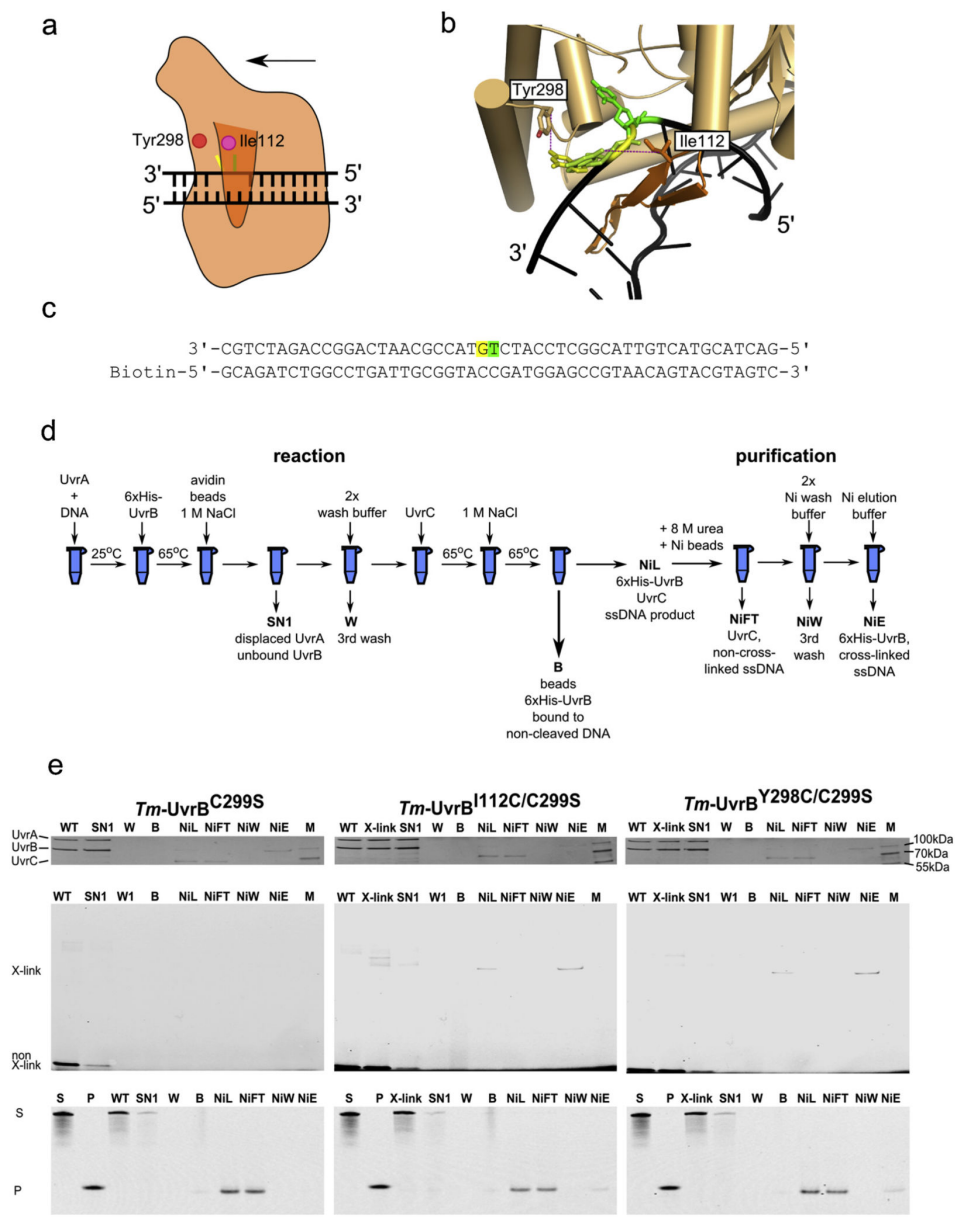
Chemical cross-linking. (a) Schematic of the cross-linking strategy for UvrB—DNA complex structure. Thiol-modified base shown in yellow. Fluorescein-modified base shown in green. β-hairpin shown in darker orange. The positions of the two residues (Ile112 and Tyr298) near the flipped bases indicated with circles. The direction of UvrB translocation on DNA shown with an arrow. (b) Close-up view of the hairpin region of UvrB in the UvrA_2_—UvrB_2_—DNA model. The β-hairpin element shown in a darker shade of orange. The thiol-modified base shown in yellow. The base rotated around the glycosidic bond shown in yellow-green. The fluorescein-modified base shown in green. Cross-linking distances shown as dashed lines. (c) Sequence of the DNA substrate (DNA^FC^) used in cross-linking experiments. Fluorescein-modified nucleotide shown in green. Thiol-modified nucleotide shown in yellow. Note: the sequence of the modified strand shown in a 3′ to 5′ direction to match the orientation in the images of the model. (d) Schematic of the experimental setup (see text and Supplementary Figures S7 and S8 for more details). ATP concentration in the experiment was 1 mM. (e) Selected fractions on silver-stained SDS-PAGE (upper panel). Selected fractions on SDS-PAGE scanned for fluorescent signals (middle panel). Analysis of selected fractions from the experiments with TBE-urea gels scanned for fluorescence (lower panel). Bands marked as X-link and non X-link represent cross-linked DNA and non-cross-linked DNA, respectively. UvrB variants used in each reaction indicated above the panels. SN1, supernatant from biotin beads; W, last wash of biotin beads; B, biotin beads boiled in sample buffer; NiL, sample loaded onto Nickel beads; NiFT, flow-through from the nickel beads; NiW, last wash of nickel beads; NiE, elution from nickel beads; M, Marker. Additional lanes on TBE-urea PAGE are S, marker for substrate; P, marker for the reaction product (12-mer DNA with fluorescein in the middle). Experiment replicated three times.

A key assumption in our model is that the modified (damaged) DNA stand is located under the β-hairpin of UvrB. To verify this, we used a thiol-based chemical cross-linking approach originally developed by Verdine and co-workers [32] and which we had previously used to map interactions within the RuvC—DNA complex [33]. According to this method, a thiol group on a two-carbon tether is introduced to the guanine base of the DNA, and a cysteine residue is introduced to the protein. If upon protein—DNA complex formation the thiol groups on the DNA and cysteine residue of the protein are located in close proximity (≤ 7 Å between the C-α of cysteine and nitrogen atom of the 2-amine group of the guanine base), then a covalent disulfide link will form. The structure of UvrB in a complex with DNA [18] and our model of UvrA—UvrB—DNA indicate that after UvrB stalls at the DNA damage (a fluorescein modification in our experimental setup), the base of the nucleotide on the 3′ side of the modified residue will reside near Tyr298 of *Tm*-UvrB and, after base rotation around the glycosidic bond, near Ile112 (Fig. 5a, b).

We predicted that the thiol group of cysteine-substituted Tyr298 or Ile112 during DNA lesion verification would form a covalent cross-link with a thiol group that is introduced to the base on the 3′ side of the lesion. So, we prepared *Tm*-UvrB variants Y298C and I112C. To prevent non-specific cross-linking, we also introduced a substitution of a nearby Cys299 to serine (C299S). In our chemical cross-linking experiments, we utilized three *Tm*-UvrB variants: *Tm*-UvrB^C299S^, *Tm*-UvrB^C299S/Y298C^ and *Tm*-UvrB^C299S/I112C^. We prepared DNA substrate (DNA^FS^) by annealing an oligonucleotide with fluorescein-modified thymine and thiol-modified guanine on its 3′ side with a complementary strand with no modifications but with biotin at its 5´ end (Fig. 5c). We first incubated DNA^FS^ with *Tm*-UvrA and *Tm*-UvrB variants to reconstitute the *Tm*-UvrB—DNA^FS^ complex and in further experiments added *Tm*-UvrC to monitor the complete incision reaction (Fig. 5d).

In the initial analysis we monitored the formation of *Tm*-UvrB—DNA^FS^ complex. To this end, we mixed DNA^FS^ with *Tm*-UvrA and *Tm*-UvrB variants and analyzed the resulting complexes by SDS-PAGE. For *Tm*-UvrB cysteine variants (*Tm*-UvrB^C299S/Y298C^, and *Tm*-UvrB^C299S/I112C^), we observed a fluorescent band when scanning SDS-PAGE for fluorescein signal. We interpreted this band as a cross-linked species of *Tm*-UvrB cysteine variant and modified DNA substrate. This band was not present when using *Tm*-UvrB^C299S^ (Fig. 5e, and Supplementary Figure S6), or when dithiothreitol (DTT) was added to the reaction at high concentrations (Supplementary Figure S6). Moreover, no fluorescent band appeared when omitting UvrA from the reaction (Supplementary Figure S6). These results confirm that the observed fluorescent bands corresponded to the specifically formed complex.

To monitor the reaction catalyzed by all the three proteins, we immobilized the DNA substrate (DNA^FC^) on streptavidin beads to perform step-wise assembly of the NER complexes (Fig. 5d, Supplementary Figure S7). After mixing the DNA^FC^ with *Tm*-UvrA protein, the *Tm*-UvrA—DNA^FC^ complex was formed. We added His-tagged UvrB variants (*Tm*-UvrB^C299S^, *Tm*-UvrB^C299S/Y298C^, and *Tm*-UvrB^C299S/I112C^) to promote dissociation of *Tm*-UvrA and formation of a stalled *Tm*-UvrB—DNA^FC^ complex, which was then immobilized on streptavidin magnetic beads. Displaced *Tm*-UvrA and excess of *Tm*-UvrB proteins were washed away, followed by adding *Tm*-UvrC to promote incisions. DNA cleavage produced a complex of *Tm*-UvrB, *Tm*-UvrC and DNA^FC^ with two nicks in one strand producing a short excised DNA fragment. Increasing the temperature to 65 °C melted the base pairing within the excised DNA fragment and promoted its dissociation as ssDNA, along with UvrB and UvrC bound to it, from the rest of the DNA which remained immobilized on the streptavidin beads. We added the supernatant with the *Tm*-UvrB—*Tm*-UvrC—ssDNA complex to Ni-NTA resin under denaturing conditions to disassemble the complex and wash away any residual, non-covalently bound DNA (Supplementary Figure S8). His-tagged UvrB bound to the resin was eluted with a buffer containing 300 mM imidazole.

We analyzed the content of the selected stages of the reaction by SDS-PAGE, stained for protein (Fig. 5e: upper panel) and scanned for fluorescence (Fig. 5e: middle panel). We ran the same samples on TBE-urea gel and scanned for fluorescence to analyze nucleic acids (Fig. 5e: lower panel). For analysis on TBE-urea gels, we also added DTT to the samples after elution from Ni-NTA resin to separate DNA from protein. The presence of a short fluorescent DNA fragment on the TBE-urea gels, corresponding to the size of the expected product in NiL fractions confirmed that the NER incision reaction successfully occurred for all three *Tm*-UvrB variants. Furthermore, fluorescent signal in NiL fractions (X-link band, Fig. 5e) could be observed on SDS-PAGE for *Tm*-UvrB^C299S/Y298C^, and *Tm*-UvrB^C299S/I112C^ because of cross-linking reaction. In the case of *Tm*-UvrB^C299S^, presence of the product band for NiL fraction in TBE-urea PAGE and absence of fluorescent X-link band in SDS-PAGE indicated that reaction took place but the cross-linking did not. These findings indicate that the substituted proteins were functional and specifically processed the substrate with fluorescein and thiol modifications. Only variants *Tm*-UvrB^C299S/Y298C^ and *Tm*-UvrB^C299S/I112C^ formed chemically cross-linked complexes.

Analysis of the TBE-urea gels showed that the final fraction that eluted from Ni-NTA resin contained the excised short fragment of DNA only in the cases with cysteine variants (Fig. 5e). Because the samples were analyzed under non-reducing and denaturing conditions this results implies that the cross-linking reaction occurred, and the DNA was covalently tethered to the protein. Importantly, our results show that DNA within the cross-linked complex can be specifically cleaved, so complexes with covalent protein-DNA link were active and showed specificity and physiological relevance. Further, SDS-PAGE revealed that the UvrB band for the two cysteine variants also had fluorescent signal (X-link band), indicating the presence of a covalently linked, fluorescent DNA fragment (Fig. 5e). As a control we prepared a UvrB variant with cysteine substitution of Val115. This residue is located on top of the hairpin, in the vicinity of the DNA, but too far to form a covalent bond with modified nucleic acid (Supplementary Figure S9a). As expected, this variant did not cross-link with the DNA (Supplementary Figure S9b), confirming the specificity of our approach. As an additional control we considered using a substrate in which the thiol-modified base would be located in the stand opposite to the fluorescein-modified strand. However, this thiol modification would also be detected as damage. The UvrA-UvrB system would thus detect both strands as damaged and UvrC incisions would be introduced to either strand, affecting the cuts in the other. This would lead to very complex results that would be difficult to interpret.

Taken together, our results indicate that the modified strand lies under the β-hairpin of UvrB. The damaged base is located right next to the hairpin on its side facing the direction of the 3′ end of the clamped strand. All these elements are consistent with our model of the damage verification step in bacterial NER, in particular UvrB scanning for the damage in 5′ to 3′ direction and stalling at the lesion (Fig. 3).

## 3 Discussion

In the present study, we elucidate the mechanism of the second step of the bacterial NER pathway. Based on EM data and independent molecular modeling, we propose that loading two UvrB molecules on two different strands of the damage-containing duplex DNA allows the pathway to break the symmetry of the initial lesion localization by UvrA and then direct cleavage by UvrC to the damaged strand. We tested our structural and mechanistic models using biochemical experiments with modified DNA duplexes. These results agree with our structural model and corroborate the mechanism of damage verification by UvrB we proposed.

UvrA is a dimeric protein that preferentially binds modified double-stranded DNA (dsDNA) [34]. The atomic structure of *T. maritima* UvrA co-crystallized with modified DNA showed that only the deformed conformation of the DNA is complementary to the UvrA surface [14]. Thus, UvrA detects the damage by recognizing the lesion-imparted DNA deformation and by inducing a conformational change in a region of the nucleic acid destabilized by the damage [14]. This indirect readout mechanism allows UvrA to recognize various DNA lesions, which explains the broad specificity of the initial damage localization in bacterial NER. The mode of UvrA action, however, poses a major challenge to bacterial NER, because only the approximate damage localization is achieved and no information is provided on which of the two DNA strands is damaged. Moreover, UvrA can also bind non-damaged DNA. This is in contrast to UvrC incisions around the damage, which are very precise, strand-specific and occur only for helix-destabilizing lesions. Therefore, the second step of bacterial NER, which involves damage verification and precise localization by UvrB, plays a key role. Nonetheless, the details of this step have been unclear and the events leading to the distinction of DNA strand with damage from undamaged DNA strand were not understood. This was mostly due to the lack of a structure of the complete UvrA—UvrB—DNA complex. To unravel the mechanism of damage verification, we examined the architecture of UvrA—UvrB—DNA complex by first modeling it and then, completely independently, determining its structure using EM. The stoichiometry of the complex determined in biochemical experiments, modeling studies and EM analysis was the same and corresponded to UvrA dimer and two UvrB monomers bound to the DNA (UvrA_2_—UvrB_2_—DNA). Thus, the UvrA dimer loads two UvrB molecules on the DNA.

Our structural modeling showed that the arrangement of the four protein subunits of the complex imposes only one physically reasonable way of connecting the DNA fragments present in the UvrA—DNA and UvrB—DNA co-crystal structures. Once these connections are made the β-hairpin in each UvrB molecule clamps the DNA strand upstream (toward the 5′ end) from the region that is bound by UvrA. Importantly, given the inherent two-fold symmetry of the complex, which was clearly demonstrated by our EM reconstruction, a different DNA strand is bound under the hairpin of each UvrB molecule in the complex.

Previously, a “padlock” model was proposed in which the β-hairpin element of UvrB clamps one of the DNA strands and is critical for DNA binding by the protein [17]. One important and contested element of the architecture of UvrA—UvrB—DNA complex was the positioning of the damaged DNA strand relative to the β-hairpin [6,22–24]. Clamping by the β-hairpin confers strong DNA binding and is crucial for UvrB function in DNA repair [35] but there was no consensus whether the damaged or non-damaged strand was clamped by the β-hairpin. To clarify this key issue, we performed site-specific thiol-based chemical cross-linking experiments which showed that the damaged strand is located under the β-hairpin of UvrB.

UvrB is a helicase structurally related to PcrA [15–17] and it possesses a 5′ to 3′ DNA unwinding activity [36]. This translocation polarity is in excellent agreement with our model of UvrA—UvrB—DNA complex. UvrB can use its translocase activity to move toward the lesion with 5′ to 3′ polarity on the damaged strand [31]. This was further corroborated in our biochemical experiments showing that for a DNA duplex with two modifications in one of the strands only the modification located closer to the 5′ end is recognized. This is because the 5′ modification is the first one encountered by UvrB during its movement in 5′ to 3′ direction on the damaged strand. This movement toward the lesion also displaces UvrA from the DNA.

UvrB has a relatively low processivity − being able to unwind ~22-27 bp of dsDNA [36]. This feature fits well the mechanism we propose. In the structural model of UvrA—UvrB—DNA complex the distance between site of UvrB loading and DNA modification is ~18 bp. Therefore, UvrB processivity would be sufficient to reach the lesion from the site of loading by UvrA. We propose that the UvrB molecule which translocates with the damaged strand under the hairpin stalls at the lesion, forming a stable nucleoprotein complex with the damage site directly before the β-hairpin. At the same time the UvrB molecule with the non-damaged strand bound under the β-hairpin, after translocating for ~22-27 nt, will dissociate from the DNA. The stalled UvrB molecule will then recruit UvrC for precise and strand-specific incisions in the DNA.

The positioning of the damaged strand under the hairpin also readily explains how UvrB stalls at the DNA lesion. The DNA under the hairpin runs through a tight channel [18]. We propose that bulky adducts, particularly base modifications, cannot be accommodated in the channel under the hairpin or in the base-binding pocket, which blocks UvrB translocation leading to the stalling of UvrB on encountering the damaged site. The translocation through smaller thymine dimers is also blocked. The clamping of the DNA with a β-hairpin motif involves flipping out of two adjacent nucleotides [18]. Thymine dimers comprise two covalently linked adjacent thymines, for which base flipping observed upon binding by UvrB and consequent wide splaying of the adjacent bases would not be possible.

Our mechanistic model of DNA damage verification explains the transition from symmetrical recognition of the lesion by dimeric UvrA to strand-specific lesion verification by UvrB, which is required to achieve precise cleavage of the damaged strand. Together with the previous description of indirect readout of DNA damage by UvrA, our results provide a key element that connects two apparently contradictory features of bacterial NER − the need to achieve very broad specificity and at the same time accurate and precise incision of the DNA. This is particularly important, because spurious DNA cleavage would lead to genomic instability rather than maintenance of genetic integrity. To provide a complete mechanistic picture of bacterial NER, future studies must elucidate the mechanism of the incision step by UvrC.

## 4 Materials and methods

### 4.1 Protein production and purification

*Thermotoga maritima* and *Thermus thermophilus* UvrA, UvrB, and UvrC proteins were overexpressed in a bacterial system from plasmids based on the pET28 vector and its modified pET28-SUMO version. UvrA protein was overexpressed at 16 °C overnight in the BL21 STAR™ (DE3) strain (Invitrogen). UvrB and UvrC proteins were overexpressed at 37 °C for 3 h in the BL21-CodonPlus(DE3)-RIL strain (Stratagene). Overexpression was induced with 0.4 mM IPTG.

UvrA and UvrC proteins were purified as 6x-His-SUMO fusion proteins, and UvrB proteins contained only the 6xHis tag. Bacterial pellets were suspended in lysis buffer (40 mM NaH_2_PO_4_ [pH 7.0], 150 mM NaCl, 5% glycerol, 10 mM β-mercaptoethanol, 1 mM phenylmethylsulfonyl fluoride, 1 μM pepstatin, 0.3 μM aprotinin, 11.7 μM leupeptin, and 1 mg/ml lysozyme). After 30-min incubation on ice, the salt concentration was increased to 1 M, and the lysate was sonicated. The lysate was centrifuged at 186,000 ×*g* for 25 min, and the supernatant was applied on nickel resin (5 ml HisTrap FF Crude, GE Healthcare). Before elution with a buffer that contained 300 mM imidazole, the resin was washed with a buffer that contained 120 mM imidazole. Next, the proteins were dialyzed against a buffer that contained 40 mM NaH_2_PO_4_ (pH 7.0), 1 M NaCl, 5% glycerol, 10 mM β-mercaptoethanol, and 60 mM imidazole. During dialysis, the proteins were digested either by Ulp1 protease (6xHis-SUMO fusions) or HRV 3C protease (UvrB proteins). Dialyzed proteins were purified on the nickel resin and then on a gel filtration column (HiLoad 16/60 Superdex 200 PG, GE Healthcare) equilibrated with 20 mM HEPES (pH 7.0), 1 M NaCl, 5% glycerol, 1 mM DTT, and 0.5 mM ethylenediaminetetraacetic acid (EDTA), pH 8.0. Proteins were stored at 4 °C or frozen in 50% glycerol.

Expression constructs for various variants of UvrB protein were obtained using QuikChange Site-Directed Mutagenesis Kit (Agilent) reactions. The primers and templates used for site directed mutagenesis for generating various mutants are listed in Supplementary Table S1. During the purification of UvrB variants for the cross-linking experiments, the protein digestion and second nickel purification steps were omitted.

### 4.2 Reconstitution of UvrA—UvrB—DNA complex

For reconstitution experiments, we used a DNA oligonucleotide, which upon self-annealing produced a substrate with 32 bp ds region and 10 nt 5′overhangs (5′-TAGTCACATCAGTGATCAGTGGTFCCGGAA CCACTGATCACT, where F is a fluorescein-modified thymine). UvrA protein (dimer) at 2.5 mg/ml was mixed with DNA at a 1:1 molar ratio. UvrB was added at two-fold molar excess over the UvrA dimer. The mixture was dialyzed for 2 h at 4 °C against 20 mM HEPES (pH 7.0), 150 mM NaCl, 5% glycerol, 1 mM DTT, and 10 mM MgCl_2_. After dialysis, adenosine diphosphate was added to a final concentration of 1 mM, and the sample was clarified through a 0.22 μm filter. The reconstituted complex was next purified by a high-performance liquid chromatography system (BioSuite High Resolution SEC Column, 250 Å, 5 μm, 7.8 mm × 300 mm, 10−500 K, Waters) attached to a MALS detector (HELEOSII/Optilab T-rEX, Wyatt). The collected fractions were run on SDS-PAGE, and fractions that corresponded to the measured mass of the complex were pooled.

### 4.3 Negative-stain EM sample preparation

A thin layer of continuous carbon (< 10 nm) was evaporated onto freshly cleaved mica sheets (EMS) using a Q150 T E carbon coater (Quorum Technologies). Carbon was incubated overnight at 50 °C before floating on 400-mesh copper grids (Agar Scientific). Prior to sample incubation, the carbon-coated grid was glow-discharged for 30 s at 45 mA using a K100X glow discharger (EMS). Four microliters of the reconstituted UvrA—UvrB—DNA complex at 15 μg/ml were applied to the glow-discharged grid and incubated for 60 s. The sample solution was partially absorbed by gentle side blotting, and the grid was immediately stained with four subsequent applications onto 50 μl drops of a 2% (w/v) uranyl formate solution, with gentle stirring for a total of 1 min. After staining, the grid was blotted dry and stored at room temperature prior to imaging.

### 4.4 Electron microscopy

UvrA—UvrB particles were imaged on a JEM-2100 LaB_6_ electron microscope (JEOL, Japan) that operated at 120 keV. A total of 423 micrographs were acquired with a 4k×4k Ultrascan CCD camera (Gatan) at a nominal magnification of 42,000× (corresponding to a pixel size of 2.73 Å at the specimen level). Images were collected in low-dose mode (electron dose of 35 e-/Å^2^),with defocus values of 0.5−2.5 μm.

### 4.5 Image processing and atomic docking

Particles were semiautomatically picked using the *e2boxer.py* program in the EMAN2 package, version 2.12 [37]. A total of 23,547 individual particles were extracted. The parameters of the Contrast Transfer Function (CTF) were estimated on each micrograph using CTFFIND3 [38]. Reference-free 2D averaging was performed using RELION, version 1.4 [39]. For 3D reconstruction of the UvrA—UvrB complex, an initial 3D volume was generated using the *sxali3d* command in SPARX [40], adopting a sphere as the starting model to minimize reference bias. The initial volume, low-pass filtered to 40 Å, was used as the reference model for 3D classification and refinement in RELION 1.4 [39]. The resolution of the refined model was estimated using the gold-standard Fourier Shell Correlation (FSC) in RELION 1.4 (0.143 cutoff) and plotted using the FSC validation server (European Bioinformatics Institute) with independently refined half-maps. The crystal structures of the UvrA—UvrB complex (PDB ID 3UWX) [7] or theoretical UvrA_2_—UvrB_2_—DNA model were used for rigid-body docking into the EM map using the *fit in map* command in UCSF Chimera [27]. The crystallographic model of the UvrA_2_—UvrB_2_ complex (PDB ID 3UWX) and independently modeled UvrA_2_—UvrB_2_—DNA complex were converted to electron density maps that were filtered at a resolution of 25 Å using the *e2pdb2mrc.py* program [37]. Two-dimensional re-projections of the generated maps were matched to 2D class averages of the UvrA—UvrB complex using the *e2classvsproj.py* program [37].

### 4.6 Modeling of the Tm-UvrA—UvrB complex

To predict the structural organization of the UvrA—UvrB—DNA complex, we first constructed homology models for UvrA and UvrB proteins. We started with the *Tm*-UvrA_2_—DNA complex structure (PDB ID 3PIH) [14], in which we retained the 32 bp DNA duplex. Based on the crystal structure of a complex of UvrA and UvrB interacting domains from *Geobacillus stearothermophilus* (PDB ID 3FPN) [19], we next predicted the structure of the *Tm*-UvrA UvrB-binding domain and added the remaining missing fragments of the *Tm*-UvrA structure (residues 61−68, 293−303, and 330−338) using the *Bs*-UvrA structure (PDB ID 2R6F) [12] as the template. The *Tm*-UvrB structural model was built using the structure of *Bacillus caldotenax* UvrB in complex with a short fragment of the DNA (PDB ID 2FDC) [18] as the template. We modeled two nucleotides missing at the 5′ end of one of the strands of the UvrB—DNA complex, to generate a “blunt” end of a 7bp DNA duplex, thus enabling connection with the DNA fragment that was bound by the UvrA subunit.

Homology models of complete *Tm-*UvrA and *Tm-*UvrB proteins were generated using the SWISS-MODEL program [41], with the exception of C-terminal helical domain IV of *Tm-*UvrB, which was folded in a template-free mode using REFINER [42].

To predict the mutual orientation of UvrA and UvrB, we used the PyRy3D program for macromolecular modeling using restraints (http://genesilico.pl/pyry3d). To account for conformational changes of UvrA and UvrB, we subdivided both proteins (with the corresponding fragments of the DNA duplex) into the following structural modules that corresponded to domains/segments that were expected to behave as independently folded, relatively rigid modules:

- Module one: UvrA residues 1−89 and 466−916 (ATP-binding domains I and II with signature domain II) plus 32 bp DNA molecule fragment and the second copy of the entire UvrA.
- Module two: UvrA residues 90−128, 250−284, and 384−465 (signature domain I).
- Module three: UvrA residues 285−383 (DNA-binding domain).
- Module four: UvrA-UvrB interacting domains − residues 129−249 of UvrA and 155−242 of UvrB.
- Module five: UvrB residues 1−154 and 243−585 with 7 bp DNA molecule fragment.

First, to model the interactions between the UvrA dimer and a single copy of UvrB, we ran a preliminary simulation with PyRy3D, starting from random positions of all components in space. The conformational space was sampled using the Monte Carlo Simulated Annealing method to minimize the number of collisions and violations of distance restraints. PyRy3D was used with the following parameters: starting temperature T_0_ = 10 in dimensionless units, temperature decrease during the simulation according to the following scheme: T_n_ = T_0_*0.999n, where n is the number of the simulation step (75 000 steps total), and a grid size of 1.0 Å. As a result, we obtained 300 models of the *Tm-*UvrA—UvrB complex. We then clustered these models and obtained three large groups of solutions (90, 37, and 24 models, respectively). Analysis of the best-scored cluster representatives revealed that only the representative of the largest cluster exhibited physically realistic orientation of DNA fragments bound to UvrA and UvrB (with relatively short gaps between the ends and with approximately coaxial orientations of helix fragments), as well as protein-protein contacts similar to those observed in the crystal structure of the UvrA—UvrB complex (PDB ID 3UWX). Therefore, this model was selected for further analyses.

To optimize the preliminary model, we ran PyRy3D again. The components only underwent limited rotations and translations (1 ° around each axis and 1.0 Å along each axis in each iteration). The scoring function parameters were set to strongly disfavor steric clashes. This time, additional distance restraints were involved that represented contacts that were previously identified by Pakotiprapha et al. [7] in the UvrA_2_—UvrB_2_ complex structure between residues Tyr203 and Glu206 of UvrB and Thr754 and Ser780 in the other subunit of the UvrA dimer. At this point, we attempted to determine the most likely distance between the UvrA- and UvrB-binding sites on the DNA and to close the gap between the DNA fragments that were included in the initial model. To achieve this, we ran multiple independent simulations, in which we kept the length of the DNA segment in the UvrB—DNA complex at 7 bp while we varied the length of DNA that was bound to the UvrA dimer by adding or removing base pairs at the end close to the UvrB-binding site. Analysis of the simulation results revealed that all restraints were fulfilled without clashes between domains and with continuous connection between the DNA strands, only for the original variant with a 32 bp DNA fragment bound to UvrA. The best-scored model from this cluster was selected for the final refinement. First, we “ligated” the ends between corresponding protein fragments and between the DNA fragments. This optimized the geometry and stereo-chemistry of the junctions using a Steepest Descent method and the AMBER force field as implemented in Hyperchem (HyperChem Professional 7.51, Hypercube, Gainesville, FL, USA). Second, we generated a nearly symmetrical *Tm*-UvrA_2_—UvrB_2_—DNA complex by copying the optimized UvrA-UvrB interface (involving the entire UvrB protein and large parts of UvrA and DNA) and replacing the second (thus far not optimized) end of the UvrA dimer.

### 4.7 Incision reaction

The incision reaction was performed in 50 mM Tris (pH 7.5) buffer with 150 mM KCl, 10 mM MgCl_2_, 5 mM DTT, and 1 mM ATP. The following protein concentrations were used for the incision reaction: 40 nM UvrA (dimer), 80 nM UvrB, and 40 nM UvrC. DNA (4 nM) was incubated with UvrA for 25 min at 25 °C followed by 50 min incubation at 65 °C along with the addition of UvrB. UvrC was then added, and the reaction was continued for 15 min under the same conditions. The reactions were stopped with sample buffer (formamide, 50 mM EDTA), heated to 95 °C for 5 min, and run on 18% TBE-urea gels (18 W, 40 min). For the radioactive experiments, DNA was labeled as described previously [14]. DNA (2 nM) and 20, 40, and 20 nM UvrA, UvrB, and UvrC, respectively, were used in the radioactive experiments. A DNA ladder was prepared by using DNA strands of appropriate lengths and either labeling them at 3′ or 5′ termini. We note the anomalous migration of the reaction products for the 3′-lableled substrate due to the presence of a fluorescein modification. We showed that such DNA migrated at the position corresponding to a ssDNA with approximately three additional nucleotides (Supplementary Figure S10).

### 4.8 Chemical cross-linking

DNA oligonucleotides that carried the 2-F-dI residue at the position of the desired through-base modification were obtained from Metabion (Martinsried, Germany) (for sequence see Fig. 5c). Synthesis columns that contained the oligonucleotides were deprotected with 3% dichloroacetic acid and washed with dichloromethane. The columns were incubated with cystamine dihydrochloride solution in trimethylamine/water (3:5) for 18 h with occasional agitation. The solution was collected from the column and dried using a speed vacuum, and the pellet was suspended in 1 ml of ammonia/40% methylamine (1:1; AMA). The columns were incubated with 1 ml of AMA solution for 2 h with occasional agitation. The washing solution was collected. Both fractions were incubated at 60 °C for 20 h and dried using a speed vacuum. The pellets were resuspended in water, pooled, and loaded on a DNA Pac100 column (Dionex) that was equilibrated with 4 M urea, 20 mM MES (pH 6.5), 1 mM NaClO_4_, and 0.2% ACN at 65 °C. Elution was performed with a linear gradient of 1−400 mM NaClO_4_. DNA from the selected fractions was retrieved by ethanol precipitation and further purified on 20% denaturing TBE-urea polyacrylamide gel. Purified oligonucleotides were stored in ultrapure water at -20 °C.

Cross-linking reactions were performed in a 600 μl volume in the reaction buffer with 50 mM Tris pH 7.5; 150 mM KCl; 10 mM MgCl_2_; 0, 0.25 or 20 mM DTT and 1 mM ATP. DNA (60 nM) was mixed with 60 nM UvrA dimer and incubated for 25 min at 25 °C. UvrB variants were then added to a concentration of 120 nM, and the reactions were divided into 100 μl aliquots. The reactions were then incubated for 1 h at 65 °C, 2 h at 37 °C, and overnight at 25 °C. Afterward, the reactions were pooled and stopped with 1 M NaCl. Next, for the immobilization of the DNA complex and reconstitution of the incision reaction, Tween-20 (0.1%) and 0.5 mg of streptavidin magnetic beads (Dynabeads MyOne Streptavidin T1, ThermoFisher Scientific) were added to the mixture. After 15 min incubation at room temperature with shaking, the beads were sedimented with a magnet and washed twice with a buffer that contained 1 M NaCl. A fresh portion of reaction buffer with 0.1% Tween-20 and 60 nM UvrC was added, and the reactions were incubated at 65 °C for 2 h with shaking. The NaCl concentration was increased to 1 M for further 15 min incubation at 65 °C. The beads were then sedimented, and the supernatant was mixed with urea to a final concentration of 8 M. The supernatant was incubated with 200 μl of 50 % nickel resin suspension (Ni-NTA agarose resin, Qiagen) for 1 h at room temperature. After two washes, bound protein was eluted with 100 μl of elution buffer that contained 300 mM imidazole. The collected fractions were kept in the dark at room temperature. We note that on the gels the bands in NiE lane are weaker in intensity than NiL because of inefficient interaction with Nickel beads as evident from the intensity of bands in the lane corresponding to NiFT.

## Supplementary Material

Supplementary Material

## Data Availability

The final UvrA—UvrB—DNA model is available at ftp://ftp.genesilico.pl/iamb/models/pyry3d(UvrAB_DNA_complex_model.pdb). The refined EM map of the UvrA—UvrB complex has been deposited in the 3D-EM database (www.emdatabank.org; accession code EMD-4958).
