# Supplementary material for: A combined structural and biochemical approach reveals translocation and stalling of UvrB on the DNA lesion as a mechanism of damage verification in bacterial nucleotide excision repair
Source: DNA Repair (Amst). Author manuscript; Available in PMC 2024 Nov 7. (PMC7616783; doi:10.1016/j.dnarep.2019.102746)
Supplement: Supplementary Material [file EMS199718-supplement-Supplementary_Material.pdf]

A combined structural and biochemical approach reveals translocation and stalling of UvrB on the DNA lesion as a mechanism of damage verification in bacterial nucleotide excision repair

Marcin Jaciuk,[1,#] Paolo Swuec,[2,#] Vineet Gaur,[1,#] Joanna M. Kasprzak,[3,4]

Ludovic Renault,[2] Mateusz Dobrychłop,[4] Shivlee Nirwal,[1]

Janusz M. Bujnicki,[3,4,]* Alessandro Costa,[2,]* Marcin Nowotny[1,]*

[1] Laboratory of Protein Structure, International Institute of Molecular and Cell Biology, Trojdena 4, Warsaw, 02-109, Poland

[2] Molecular Machines Laboratory, The Francis Crick Institute, London, NW1 1AT, UK

[3] Laboratory of Bioinformatics and Protein Engineering, International Institute of Molecular and Cell Biology, Trojdena 4, Warsaw, 02-109, Poland

[4] Institute of Molecular Biology and Biotechnology, Faculty of Biology, Adam Mickiewicz University, ul. Umultowska 89, Poznan, 61-614, Poland

[#] These authors contributed equally
* Correspondence: Marcin Nowotny, Alessandro Costa, Janusz M. Bujnicki

SUPPLEMENTARY INFORMATION

SUPPLEMENTARY RESULTS

Verification of the activity of UvrA-UvrB complex

The reconstituted UvrA—UvrB—DNA complex contained a heterologous combination of *Tm*-UvrA and *Tt*-UvrB. We wished to verify whether this combination is functional in a NER reaction. To test this, we reconstituted NER incision *in vitro* with purified recombinant *Tm*-UvrA, *Tm*-UvrB, and *Tm*-UvrC proteins or a combination of *Tm*-UvrA, *Tt*-UvrB, and *Tm*-UvrC proteins. For the substrate, we used a previously published 50-mer dsDNA with single fluorescein-modified thymine (F26) (Jiang et al., 2006) and analyzed the reaction products on Tris/Borate/EDTA (TBE)-urea PAGE. We exploited the fact that the excised fragment contained fluorescein, so we used fluorescence for DNA visualization. The results showed that the combination of *Tm*-UvrA, *Tm*-UvrB, and *Tm*-UvrC efficiently excised a 12 nt DNA fragment at an optimal temperature of 65°C (Supplementary Figure S1c). This fragment resulted from two cuts mediated by UvrC: one cut occurred 7 nt from the lesion (the position of fluorescein-modified thymine) on its 5′ side, and the other cut occurred 4 nt from the lesion on the 3′ side. When we exchanged *Tm*-UvrB for *Tt*-UvrB in the reaction, we obtained the same product, though with slightly lower efficiency. This result confirms that the interspecies combination was functional.

Jiang, G.H., Skorvaga, M., Croteau, D.L., Van Houten, B., and States, J.C. (2006). Robust incision of Benoz[a]pyrene-7,8-dihyrodiol-9,10-epoxide-DNA adducts by a recombinant thermoresistant interspecies combination UvrABC endonuclease system. Biochemistry *45*, 7834-7843.

SUPPLEMENTARY FIGURES AND TABLE

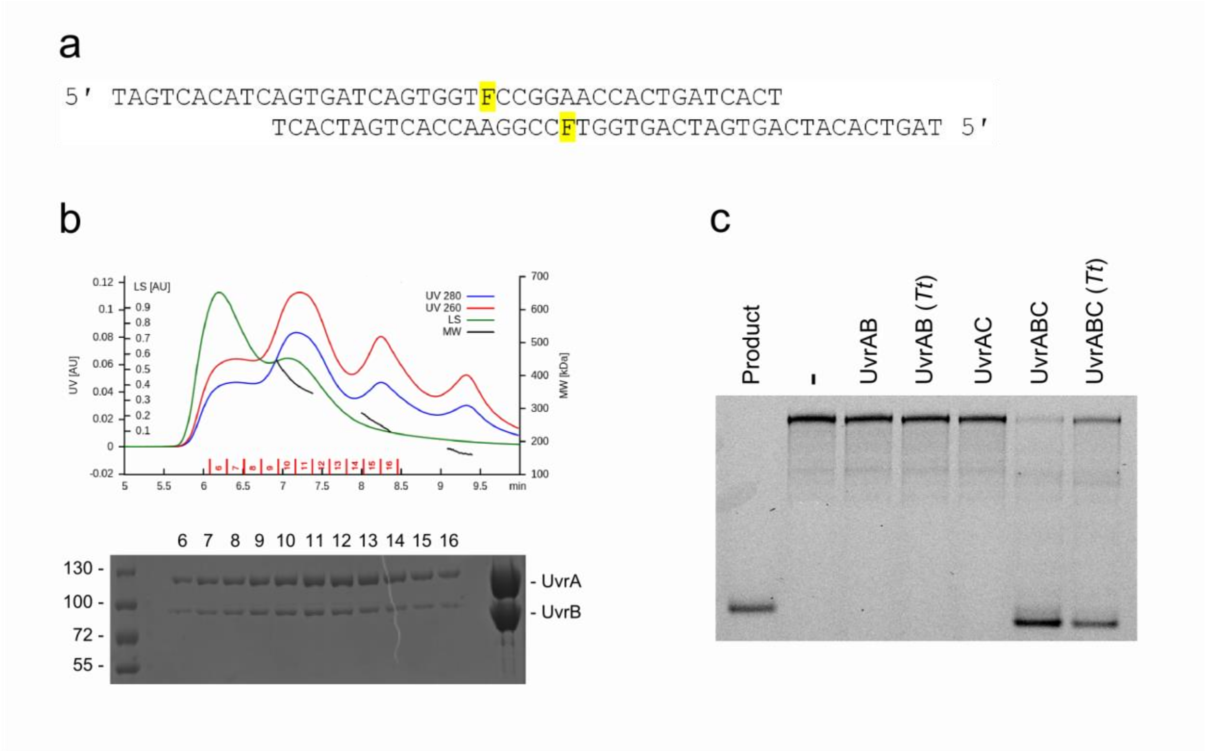

Supplementary Figure S1. Reconstitution of UvrA—UvrB-DNA complex. (a) Schematic of the self-annealing DNA substrate used for complex reconstitution. ‘F’ highlighted in yellow is the position of fluorescein-modified thymine. Reconstitution of the complex (SEC experiment, BioSuite High Resolution SEC Column). The absorbance values at 280 and 260 nm are shown as blue and red traces, respectively. The green trace is the static light scattering, and black lines correspond to the molecular weight determined from the multiple-angle light scattering measurement (right axis). The content of the fractions was analyzed by SDS-PAGE (lower panel). The numbers identify the fractions in the elution profile above. This experiment was replicated two times. **(b)** Incision assay for interspecies protein combination. 50-mer DNA substrate contained a fluorescein modification in the middle, which both served as the modification that was processed by NER and was used to

visualize the product. The DNA was mixed with NER proteins indicated on top of each lane. UvrABC corresponds to *T. maritima* proteins and (*Tt*) indicates that *T. thermophilus* UvrB was used in the reaction. This experiment was replicated two times.

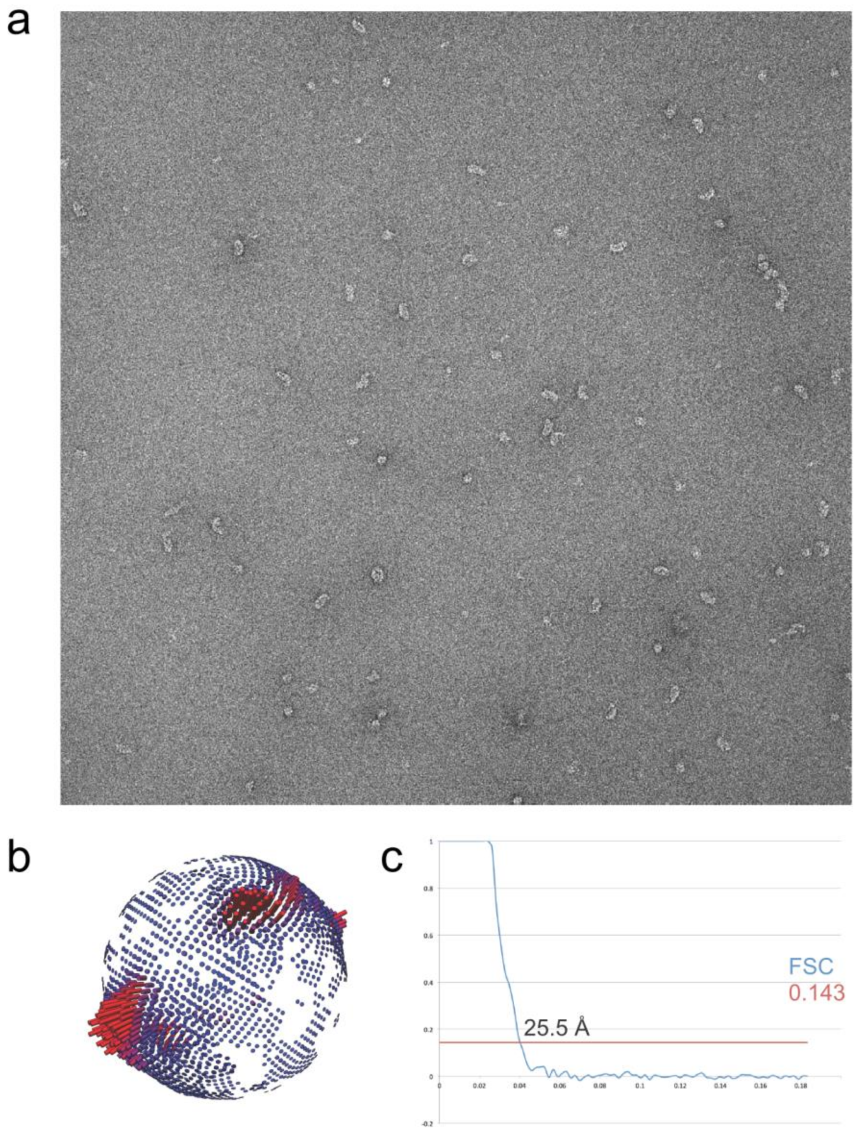

Supplementary Figure S2. Single-particle negative-stain electron microscopy of the UvrA—UvrB—DNA complex structure. (a) Representative negatively stained micrograph. **(b)** Angular distribution. **(c)** Gold-standard Fourier shell correlation (FSC) and resolution according to the 0.143 criterion.

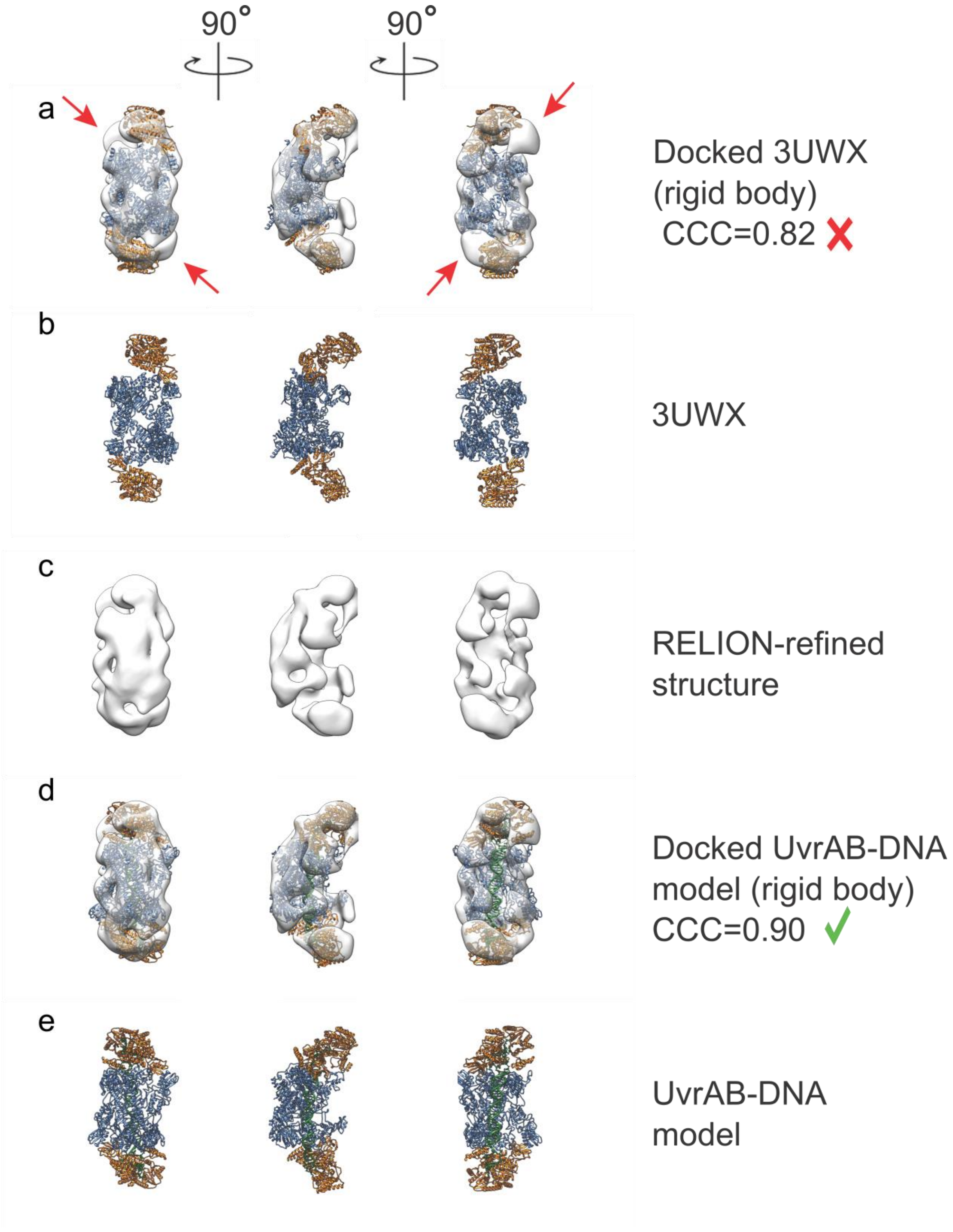

Supplementary Figure S3. Comparison between the EM structure and two alternative atomic models for $UvrA_2$—$UvrB_2$. (a) UvrA-UvrB crystal structure (PDB ID 3UWX) docked into the EM density map (CCC = 0.82). Red arrows indicate unoccupied electron density.

(b) Crystal structure of the UvrA—UvrB complex (PDB ID 3UWX). **(c)** 3D EM structure of $UvrA_2$—$UvrB_2$—DNA complex refined with RELION. **(d)** Independently modeled $UvrA_2$—$UvrB_2$—DNA complex docked into the EM density map (CCC = 0.9). When the CCC was computed, only the protein component of the modeled structure was used. **(e)** Model of the $UvrA_2$—$UvrB_2$—DNA complex.

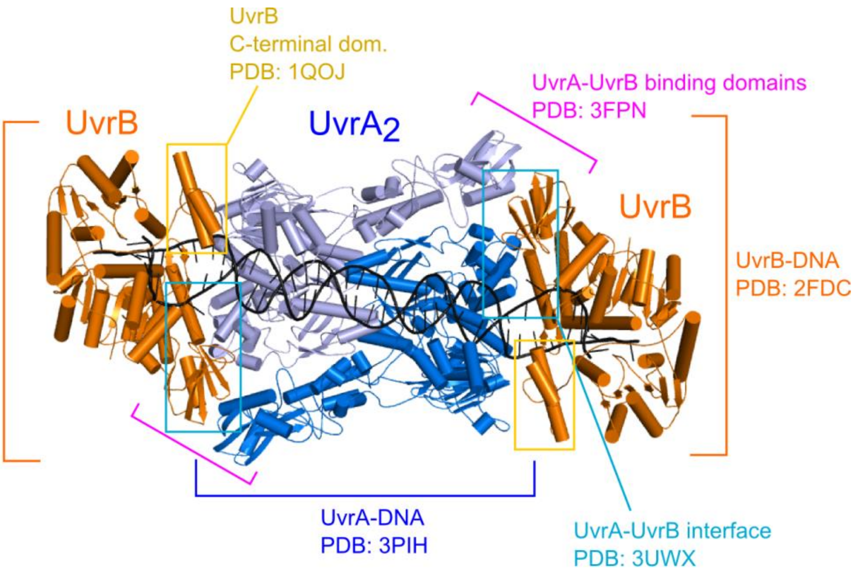

Supplementary Figure S4. Crystal structures used in the modeling of the UvrA—UvrB—DNA complex.

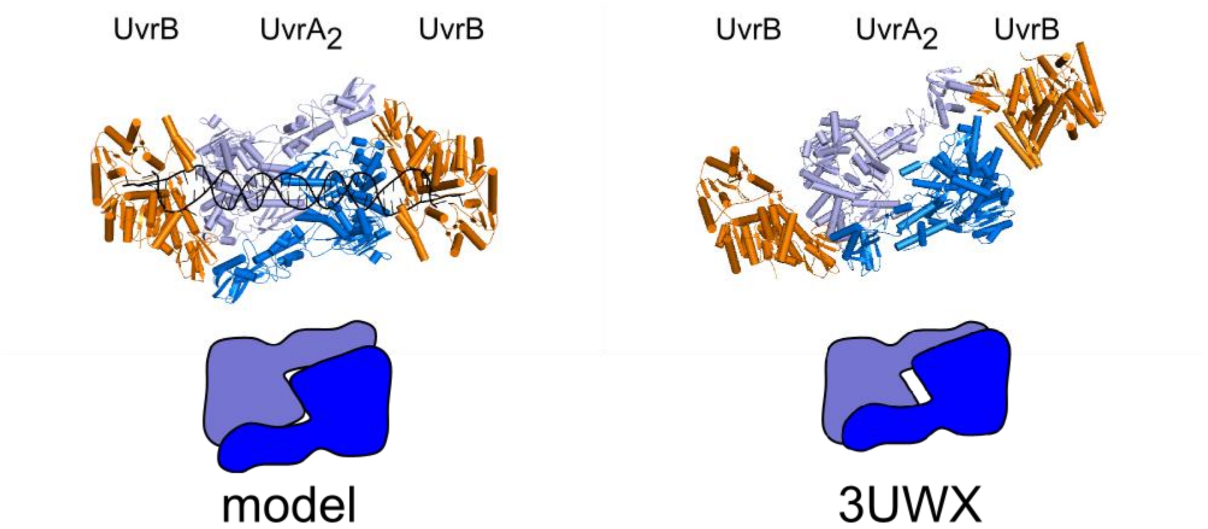

Supplementary Figure S5. Comparison of the UvrA—UvrB—DNA model (present study) with a crystal structure of the UvrA—UvrB complex (PDB ID 3UWX).

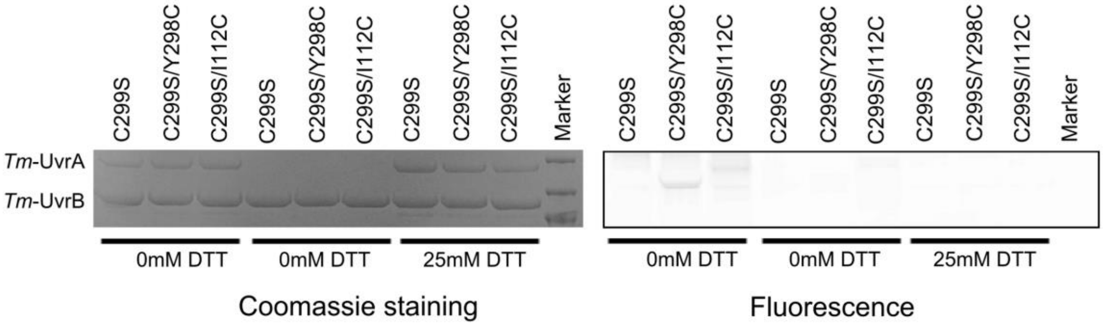

Supplementary Figure S6. Chemical cross-linking. Results of cross-linking experiments. UvrA and UvrB were mixed with modified DNA (DNA^{FS}) in the presence of ATP. The reaction products were resolved by SDS-PAGE stained with silver (left panel) or visualized for the fluorescence of cross-linked DNA (right panel). The reactions were either in the presence (left lanes and right lanes) or absence of *Tm*-UvrA (middle lanes). This experiment was repeated five times.

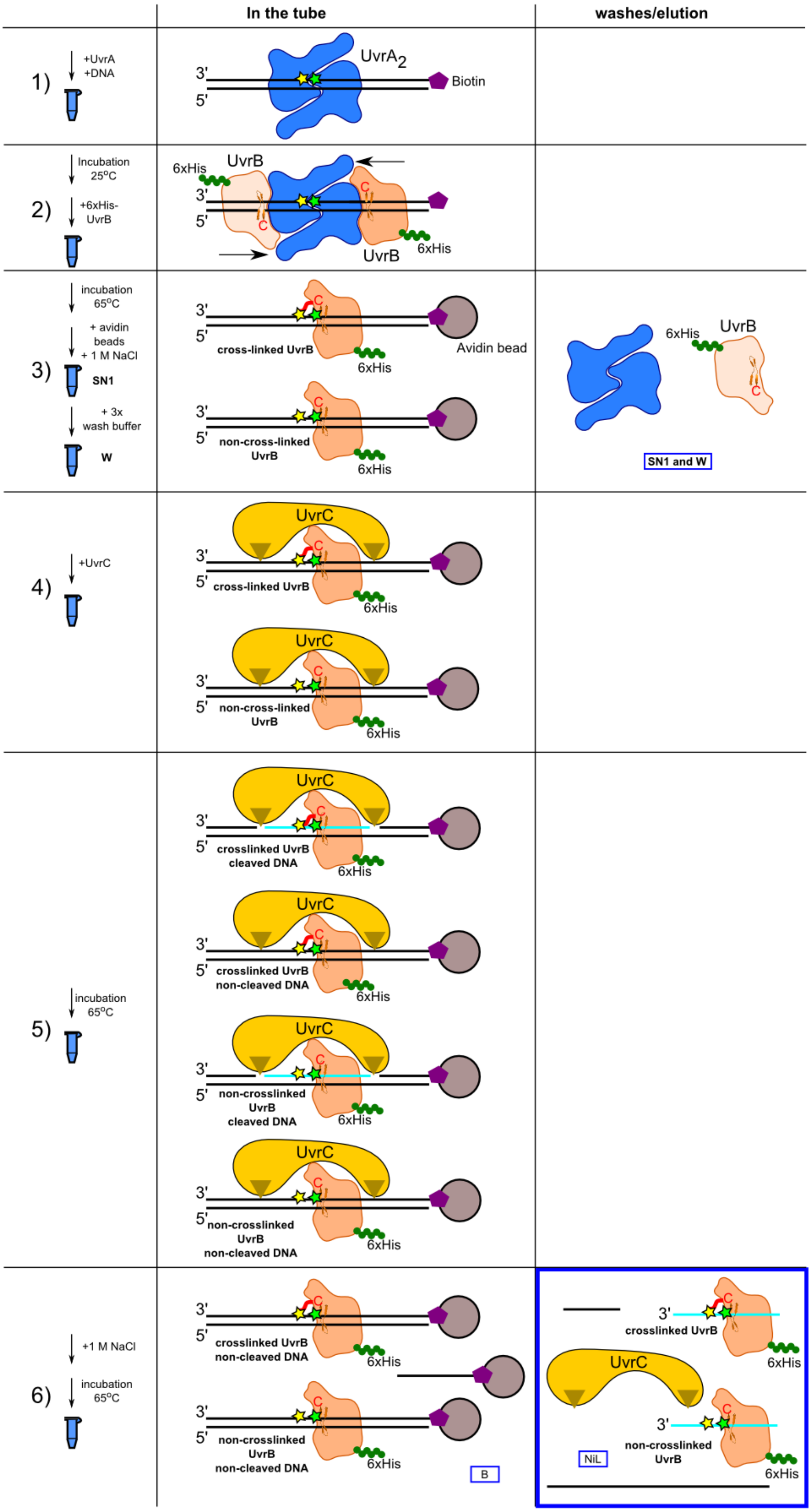
In the tube
washes/elution
1)
+UvrA
+DNA
UvrA2
3'
5'
Biotin
2)
Incubation
25°C
+6xHis-
UvrB
6xHis
UvrB
C
3)
incubation
65°C
+ avidin
beads
+ 1 M NaCl
SN1
+ 3x
wash buffer
W
cross-linked UvrB
Avidin bead
non-cross-linked
UvrB
SN1 and W
4)
+UvrC
UvrC
5)
incubation
65°C
crosslinked UvrB
cleaved DNA
crosslinked UvrB
non-cleaved DNA
non-crosslinked
UvrB
cleaved DNA
non-crosslinked
UvrB
non-cleaved DNA
6)
+1 M NaCl
incubation
65°C
B
NiL

Supplementary Figure S7 (previous page). **Schematic of the cross-linking reaction.** In step 1) UvrA (blue) was mixed with the DNA (fluorescein position indicated as a green star and thiol modification position as a yellow star, 5′ biotin in purple). In step 2), after incubation at 25°C to allow UvrA-DNA complex formation, His-tagged UvrB was added (yellow). In step 3) incubation at 65°C in the presence of ATP allowed UvrB loading and translocation toward the lesion. Next, avidin beads (gray circle) were added, supernatant (SN1) was collected and the beads were washed three times (last wash W was collected). UvrA and UvrB molecule in lighter orange dissociated and were present in SN1 and W. The position of cysteine introduced to UvrB is marked as red "C". Disulfide cross-link is shown as a red wavy line. Cross-linking reaction is not 100% efficient and thus both cross-linked and non-cross-linked UvrB species were present on the beads. In step 4) the reaction was supplemented with UvrC which interacted with UvrB both in the cross-linked and non-cross-linked form. In Step 5) the reaction was incubated at 65°C to allow incisions by UvrC. The cleaved DNA fragment is shown in cyan. Because this reaction is not 100% efficient both incised and non-incised DNAs were present. The incisions could occur both in cross-linked and non-cross-linked complexes leading to the formation of four possible types of complexes. In step 6) buffer with 1 M NaCl was added to promote dissociation of UvrC and the reaction was further incubated at 65°C to promote melting of base pairs in the short DNA fragment generated by UvrC incisions. Non-incised DNA and associated proteins remained on the beads. Eluted fraction thus contained UvrC, short DNA and UvrB in complex with cross-linked and non-crosslinked DNA. This fraction (boxed in blue) was used in the purification step (Supplementary Figure S8). Names of the fractions used in Figure 5 are shown in blue boxes.

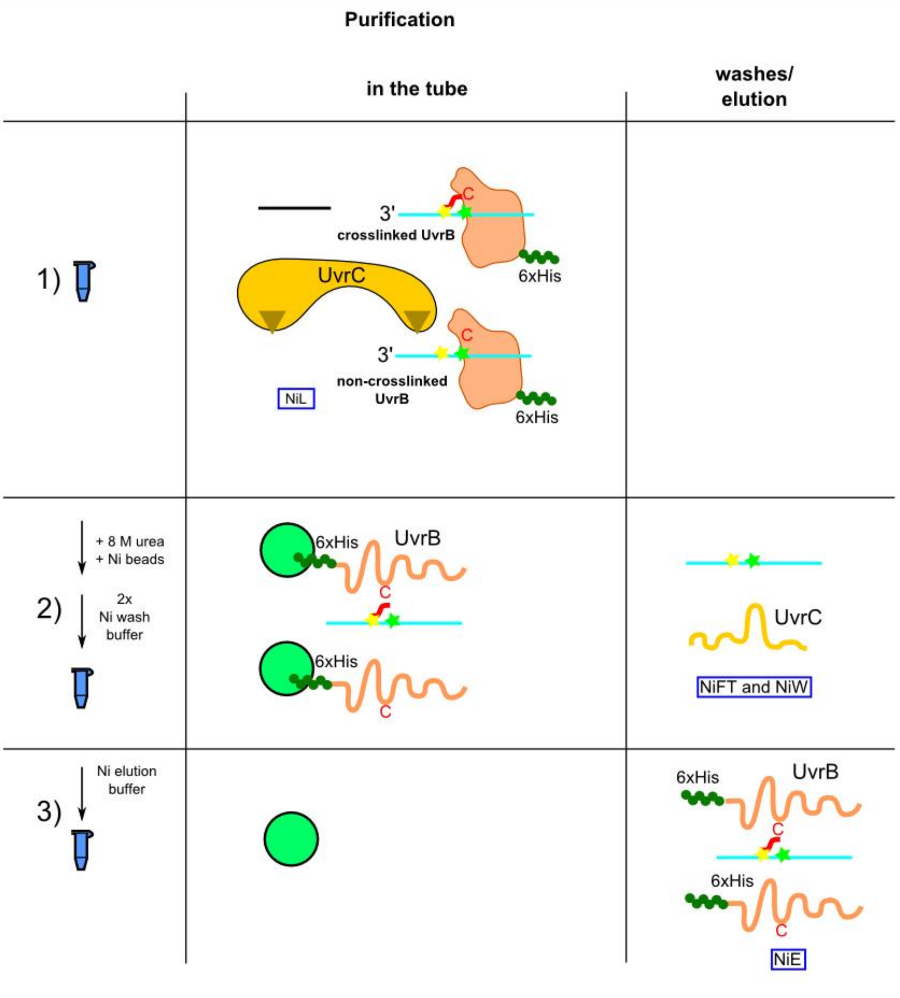

Supplementary Figure S8. **Schematic of the cross-linking purification.** The color scheme as in Supplementary Figure S7. Elution from the cross-linking reaction (Supplementary Figure S7, blue box) was mixed with 8 M urea to denature the proteins and disrupt any non-covalent complexes. Next, Ni beads were added. In step 2) UvrB (both crosslinked to the DNA and without the DNA) bound to the beads. Denatured UvrC and non-crosslinked DNA were present in the flow-through (FT) and washes (NiW). In step 3) UvrB was eluted from the Ni beads (NiE). Names of the fractions used in Figure 5 are shown in blue boxes.

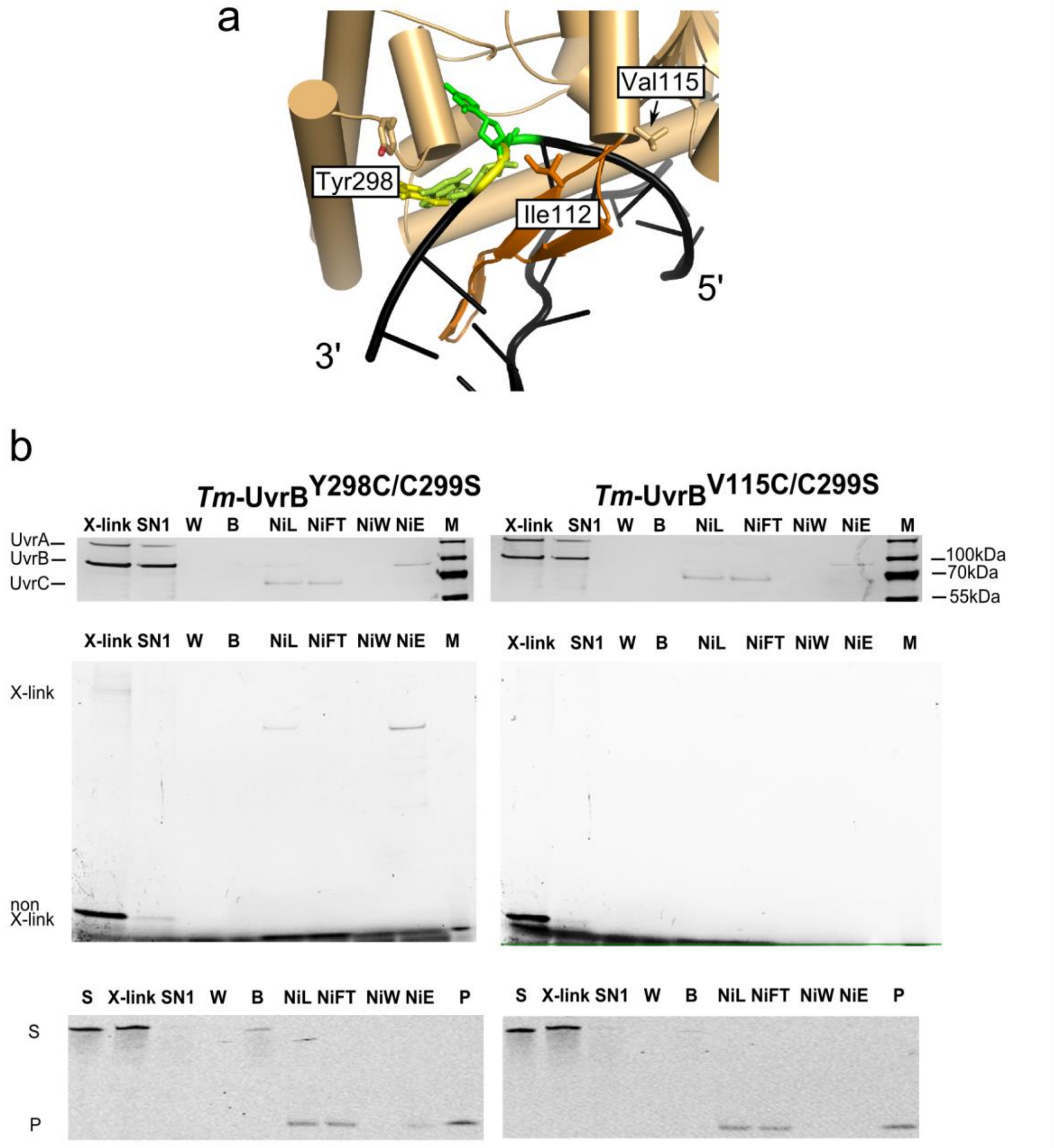

Supplementary Figure S9. Chemical cross-linking. (a) Close-up view of the hairpin region of UvrB in the $UvrA_2$—$UvrB_2$—DNA model. The β-hairpin element shown in a darker shade of orange. The thiol-modified base shown in yellow. The base rotated around the glycosidic bond shown in yellow-green. The fluorescein-modified base shown in green. The residues individually substituted to cysteine are shown as sticks and labeled. Val115 substituted in this control experiment is indicated with an arrow. **(b)** Selected fractions on silver-stained SDS-PAGE (upper panel). Selected fractions on SDS-PAGE scanned for fluorescent signals

(middle panel). Analysis of selected fractions from the experiments with TBE-urea gels scanned for fluorescence (lower panel). Bands marked as X-link and non X-link represent cross-linked DNA and non-cross-linked DNA, respectively. UvrB variants used in each reaction indicated above the panels. SN1, supernatant from biotin beads; W1, last wash of biotin beads; B, biotin beads boiled in sample buffer; NiL, sample loaded onto Nickel beads; NiFT, flow-through from the nickel beads; NiW, last wash of nickel beads; NiE, elution from nickel beads; M, Marker. Additional lanes on TBE-Urea page are S, marker for substrate; P, marker for the reaction product (12-mer DNA with fluorescein in the middle). Experiment replicated three times.

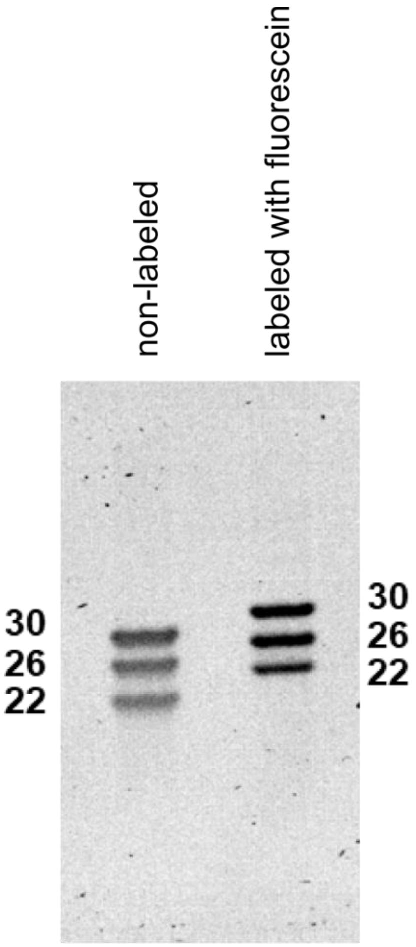

Supplementary Figure S10. Comparison of migration of fluorescein-modified oligonucleotides with their non-labeled counterparts. The DNAs were resolved on TBE-urea gel stained with SYBR gold.

Supplementary Table S1: Primers and templates used for generating various mutants of *Tm*-UvrB (written 5′ to 3′)

	Mutant	Template	Primers used for Quikchange site-directed mutagenesis
1	*Tm*-UvrB^{C299S}	*Tm*-UvrB^{WT}	GTTCTCTATTCCAGGACTGTATCCCATGGTCTCCA
			TGGAGACCATGGGATACAGTCCTGGAATAGAGAAC
2	*Tm*-UvrB$^{C299S/Y298C}$	*Tm*-UvrB^{C299S}	CTCTATTCCAGGACAGCATCCCATGGTCTCCAGC
			GCTGGAGACCATGGGATGCTGTCCTGGAATAGAG
3	*Tm*-UvrB$^{C299S/I112C}$	*Tm*-UvrB^{C299S}	TCGCACGATCACATCGTTGCAGTCAGCGTTCTTCTCTATG
			CATAGAGAAGAACGCTGACTGCAACGATGTGATCGTGCGA
4	*Tm*-UvrB$^{C299S/V115C}$	*Tm*-UvrB^{C299S}	CATTCTCATTCGCACGATGCAATCGTTGATGTCAGCGTTCTTCTCTAT
			ATAGAGAAGAACGCTGACATCAACGATTGCATCGTGCGAATGAGAATG
